# Association of Genes Involved in the Metabolic Pathways of Amyloid-β and Tau Proteins With Sporadic Late-Onset Alzheimer’s Disease in the Southern Han Chinese Population

**DOI:** 10.3389/fnagi.2020.584801

**Published:** 2020-11-06

**Authors:** Xuewen Xiao, Bin Jiao, Xinxin Liao, Weiwei Zhang, Zhenhua Yuan, Lina Guo, Xin Wang, Lu Zhou, Xixi Liu, Xinxiang Yan, Beisha Tang, Lu Shen

**Affiliations:** ^1^Department of Neurology, Xiangya Hospital, Central South University, Changsha, China; ^2^National Clinical Research Center for Geriatric Disorders, Central South University, Changsha, China; ^3^Key Laboratory of Hunan Province in Neurodegenerative Disorders, Central South University, Changsha, China; ^4^Department of Geriatrics Neurology, Xiangya Hospital, Central South University, Changsha, China; ^5^Department of Radiology, Xiangya Hospital, Central South University, Changsha, China; ^6^Key Laboratory of Organ Injury, Aging and Regenerative Medicine of Hunan Province, Changsha, China

**Keywords:** Alzheimer’s disease, Chinese, amyloid-β, tau, metabolism

## Abstract

The genes involved in the metabolic pathways of amyloid-β (Aβ) and tau proteins significantly influence the etiology of Alzheimer’s disease (AD). Various studies have explored the associations between some of these genes and AD in the Caucasian population; however, researches regarding these associations remain limited in the Chinese population. To systematically evaluate the associations of these genes with AD, we investigated 19 genes involved in the metabolism of Aβ and tau based on previous studies selected using the PubMed database. This study included 372 patients with sporadic late-onset AD (sLOAD) and 345 cognitively healthy individuals from southern China. The results were replicated in the International Genomics of Alzheimer’s Project (IGAP). Protein–protein interactions were determined using the STRING v11 database. We found that a single-nucleotide polymorphism, rs11682128, of *BIN1* conferred susceptibility to sLOAD after adjusting for age, sex, and *APOE* ε4 status and performing the Bonferroni correction {corrected *P* = 0.000153, odds ratio (OR) [95% confidence interval (CI)] = 1.403 (1.079–1.824)}, which was replicated in the IGAP. Protein–protein interactions indicated that BIN1 was correlated with MAPT. Moreover, rare variants of *NEP* and *FERMT2* (0.0026 < corrected *P* < 0.05), and the Aβ degradation, tau pathology, and tau phosphatase pathways (0.01 < corrected *P* < 0.05), were nominally significantly associated with sLOAD. This study suggested that the genes involved in the metabolic pathways of Aβ and tau contributed to the etiology of sLOAD in the southern Han Chinese population.

## Introduction

Alzheimer’s disease (AD) is the most common neurodegenerative disorder characterized by cognitive impairment and neuropsychiatric symptoms (Scheltens et al., 2016). The prevalence of AD is rising rapidly owing to the aging of the populations. It is estimated that 50 million individuals have dementia worldwide, two thirds of whom are diagnosed with AD (Saez-Atienzar and Masliah, 2020). The global incidence of patients with dementia is expected to reach 152 million by 2050 (Patterson, 2018). AD can be classified as early onset AD (EOAD) (age of onset <65 years) or late-onset AD (LOAD) (age of onset ≥65 years), depending on the age at the time of onset (Li et al., 2018). Moreover, AD can be divided into familial AD (FAD) and sporadic AD based on family history (Dorszewska et al., 2016). The term sporadic LOAD (sLOAD) is used to describe sporadic AD patients with an onset age ≥65 years (Cruchaga et al., 2018).

Amyloid precursor protein (*APP*), presenilin 1 (*PSEN1*), and presenilin 2 (*PSEN2*) are causative genes associated with FAD (Jiao et al., 2014). However, the etiology of sLOAD remains elusive. Multiple genetic and environmental risk factors contribute to the pathogenesis of sLOAD. To date, more than 40 risk loci for AD have been identified by genome-wide association studies (GWASs), which have implicated amyloid-β (Aβ), tau, and lipid metabolism in the development of AD (Lambert et al., 2013; Kunkle et al., 2019). Aβ and tau protein deposition are the two principal pathological hallmarks of AD. The imbalance between Aβ production and clearance leads to the aggregation of senile plaques, which result in neuronal loss in AD (Hardy and Selkoe, 2002). Meanwhile, the hyperphosphorylated tau protein can transform into neurofibrillary tangles under pathological conditions, also causing neuronal degeneration in AD. Thus, the metabolism of Aβ and tau proteins plays a critical role in the pathogenesis of AD (Kametani and Hasegawa, 2018).

Amyloid-β is generated by the proteolytic cleavage of the Aβ protein precursor by β-secretases and γ-secretases. The beta-amyloid cleaving enzyme 1 (BACE1) is a β-secretase enzyme, which initiates the cleavage of APP to form Aβ. A recent study showed that the beta-amyloid cleaving enzyme 2 (BACE2), a homolog of BACE1, can also process APP at the β-site and contribute to the pathogenesis of AD (Wang et al., 2019). The Aβ clearance rate was significantly impaired in patients with LOAD compared with cognitively healthy individuals, while no differences in the Aβ production rate were observed between the two groups (Mawuenyega et al., 2010). Thus, the clearance of Aβ is of great importance in the pathogenesis of LOAD. Aβ-degrading proteases are involved in the proteolytic degradation of Aβ, including neprilysin (NEP), endothelin-converting enzyme-1 (ECE-1), insulin-degrading enzyme (IDE), membrane metallo-endopeptidase-like 1 (MMEL1), angiotensin-converting enzyme (ACE), and matrix metalloproteinase-9 (MMP-9) (Saido and Leissring, 2012).

The equilibrium of tau phosphorylation is regulated by tau kinases and phosphatases (Oliveira et al., 2017). Tau kinases contribute to the phosphorylation of tau protein by transferring the phosphate group from high-energy donor molecules to the tau protein. Common tau kinases include glycogen synthase kinase-3 β (GSK3β), cyclin-dependent protein kinase-5 (CDK5), and mitogen-activated protein kinase 1 (MAPK1) (Martin et al., 2013). Phosphorylated tau proteins can be dephosphorylated by tau protein phosphatases. Protein phosphatase-2A (PP2A) is one of the primary tau phosphatases associated with decreased Aβ production in the human brain (Shentu et al., 2018). The overall activity of PP2A is facilitated by holoenzymes consisting of a catalytic subunit alpha (PP2CA) and a regulatory subunit B alpha (PP2R2A). Its activity is inhibited by the acidic nuclear phosphoprotein 32 family member A (ANP32A), which acts as an inhibitor. The methylation of PP2A by leucine carboxyl methyltransferase-1 (LCMT1) can increase PP2A activity (Stanevich et al., 2011); in contrast, demethylation by protein phosphatase methylesterase-1 (PPME1) reduces its activity (Liu et al., 2016). Peptidyl-prolyl *cis*-*trans* isomerase NIMA-interacting 1 (PIN1) induces conformational changes in tau protein and promotes tau dephosphorylation by PP2A (Vázquez-Higuera et al., 2011). Additionally, fermitin family member 2 (FERMT2) and bridging integrator 1 (BIN1) are involved in the modulation of tau pathology in both *Drosophila* and humans (Chapuis et al., 2013; Farfel et al., 2016).

**FIGURE 1:**
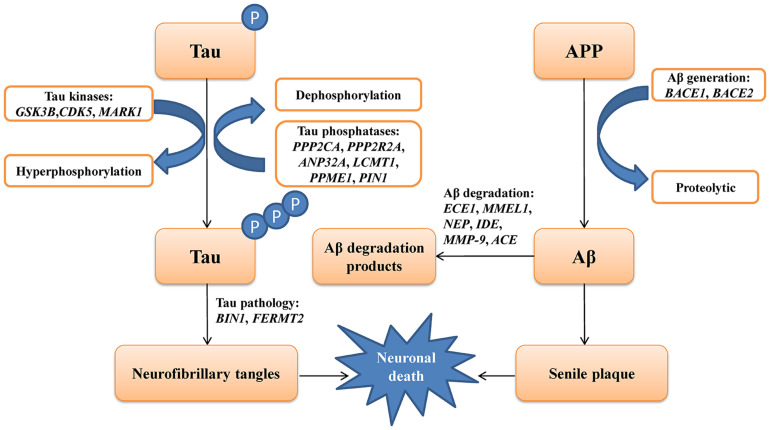
A schematic picture showing the genes involving the metabolic pathways of amyloid-β (Aβ) and tau protein in Alzheimer’s disease.

The genes involved in the metabolic pathways of Aβ and tau have been reported to play a critical role in AD. Various studies have explored the association between some of these genes and AD. However, most of these studies were conducted in the Caucasian population (Clarimon et al., 2003; Helisalmi et al., 2004; Blomqvist et al., 2010), whereas data regarding the Chinese population remain limited (Zhou et al., 2018; Zhang et al., 2019b). In this study, we selected 19 genes involved in the metabolism of Aβ and tau, including Aβ degradation (*ECE1*, *MMEL1*, *NEP*, *IDE*, *MMP9*, and *ACE*), Aβ generation (*BACE1* and *BACE2*), tau kinases (*GSK3B*, *CDK5*, and *MARK1*), tau pathology (*FERMT2* and *BIN1*), and tau phosphatases (*PPP2CA*, *PPP2R2A*, *ANP32A*, *LCMT1*, *PPME1*, and *PIN1*) based on previously published studies available in the PubMed database (Figure 1). These five different metabolic pathways of genes associated with Aβ and tau proteins were screened in 372 patients with sLOAD and 345 cognitively healthy individuals. The single common variant association test and cumulative rare variant association test (gene and pathway based) were conducted for patients with sLOAD and cognitively healthy individuals [common variants: minor allele frequency (MAF) > 0.05; rare variant: MAF ≤ 0.05].

## Materials and Methods

### Participants

We recruited 372 patients with sLOAD (mean age at onset was 72.42 ± 7.82 years) from the outpatients and inpatients of the Department of Neurology, Xiangya Hospital, Central South University, and 345 cognitively healthy individuals (mean age at onset was 70.58 ± 5.34 years) from a community in Changsha. Mini-Mental State Examination (MMSE) scores were used to assess the cognitive ability of the cognitively healthy individuals. Their MMSE scores were equal or above 26 points (Table 1). The sLOAD patients met the National Institute on Aging/Alzheimer’s Association criteria for probable AD (McKhann et al., 2011). The courses of cognitive impairment for patients with sLOAD were over 6 months. The scores of the modified Hachinski ischemia scale were all less than 4. All selected participants were of southern Han Chinese descent, mainly from the provinces of Hunan, Jiangxi, Guizhou, and Guangxi provinces. Participants with causative mutations for AD (*APP*, *PSEN1*, and *PSEN2*) were excluded by Sanger sequencing. Additionally, we did not enroll the AD patients with secondary AD causes, such as vitamin deficiency, hypothyroidism, HIV, and syphilis infection. Patients with cerebral diseases and other neurological diseases were also excluded. This study was approved by the Ethics Committee of Xiangya Hospital of the Central South University (equivalent to an institutional review board). Written informed consent was obtained from each participant or their legal representatives.

**TABLE 1:** Demographic data and neuropsychological assessment of the subjects.

	sLOAD	Cognitively healthy individuals	*P*
Age, mean ± SD	72.42 ± 7.82	70.58 ± 5.34	0.00028^a^
Gender, male/female	143/229	165/180	0.01120^b^
MMSE, mean ± SD	11.31 ± 6.96	28.49 ± 1.22	0.00000^a^
MoCA, mean ± SD	6.41 ± 5.92	NA	NA
ADL, mean ± SD	20.24 ± 21.99	NA	NA
NPI, mean ± SD	14.39 ± 17.43	NA	NA
CDR, mean ± SD	0.69 ± 1.05	NA	NA
APOE ε4 status, APOE ε4^+^ carrier/total participants	152/372 (40.9%)	69/345 (20%)	0.0000^b^

*sLOAD, sporadic late-onset Alzheimer’s disease; APOE, apolipoprotein E; SD, standard deviation; NA, not available; MMSE, Mini-Mental State Examination; MoCA, Montreal Cognitive Assessment; ADL, activities of daily living; NPI, Neuropsychiatric Inventory; CDR, Clinical Dementia Rating.*

*^*a*^*P* value was calculated by Mann–Whitney *U* test.*

*^*b*^ P value was calculated by chi-square test.*

### Gene Screening and Genotyping

Genomic DNA was extracted from the peripheral blood leukocytes of each participant using the QIAGEN kit according to the manufacturer’s instructions. The quality and quantity of DNA were assessed using a fluorometer. All DNA samples were normalized to 50–100 ng/μl. We designed a targeted panel that included the following genes: Aβ degradation (*ECE1*, *MMEL1*, *NEP*, *IDE*, *MMP9*, and *ACE*) (Natunen et al., 2012; Baranello et al., 2015), Aβ generation (*BACE1* and *BACE2*) (Wang et al., 2019), tau kinases (*GSK3B*, *CDK5*, and *MARK1*) (Martin et al., 2013), tau pathology (*FERMT2* and *BIN1*) (Shulman et al., 2014; Calafate et al., 2016), and tau phosphatases (*PPP2CA*, *PPP2R2A*, *ANP32A*, *LCMT1*, *PPME1*, and *PIN1*) (Vázquez-Higuera et al., 2011). The participants’ genomic DNA was fragmented into 150–200 base-pair long fragments using the Bioruptor Pico (Belgium). Subsequently, the fragmented DNA was subjected to end-repairing, A-tailing, adaptor ligation, and an 11-cycle pre-capture polymerase chain reaction (PCR) amplification. After the PCR amplification, the DNA fragments were captured by the targeted panel, which was followed by sequencing using the Illumina NovaSeq 6000 platform. The mean sequencing depth was 641.6×, and the average sample coverage was 99.94%. The proportion of target region that covered ≥20× (%) and ≥30× (%) was 98.76% and 97.51%, respectively. The reads were mapped to the human reference genome (UCSC hg19/GRCH37) using the BWA software (version 0.7.15)^1^ (Li and Durbin, 2010), and duplicate sequence reads were removed by using Picard (version 2.18.7)^2^. Variant calling was conducted using the Genome Analysis Toolkit (GATK) (version 3.2)^3^ (McKenna et al., 2010). The variants were annotated using ANNOVAR^4^ (Wang et al., 2010) and named according to the guidelines of the Human Genome Variation Society (HGVS)^5^ (den Dunnen et al., 2016).

### Statistical Analyses

#### Common Variant Association Test

Variants with MAF ≤ 0.05, genotyping rate <80%, and Hardy–Weinberg *P* value < 0.001 were filtered out using PLINK 1.9 (Purcell et al., 2007). For the remaining common variants, the association test was performed between sLOAD patients and cognitively healthy individuals by using PLINK 1.9. Furthermore, age, sex, and *APOE*ε4 status (*APOE*ε4+ and *APOE*ε4−) were adjusted for each common variant with PLINK 1.9. Linkage disequilibrium (LD) patterns of the genes with nominally significant variants were reconstructed using Haploview version 4.2 (Barrett et al., 2005). Additionally, we performed the meta-analysis of nominally significant variants from our study and reported studies using the RevMan 5.4 software^6^.

#### Gene-Based Association Test

The optimized sequence kernel association test (SKAT-O) (Lee et al., 2012), which optimally combined the SKAT and burden tests, was installed in R using SKAT v1.0.9 to compare the aggregate burden of each gene between sLOAD patients and cognitively healthy individuals. The rare variants (MAF ≤ 0.05) within each gene were collapsed together, and their effects on sLOAD were assessed using the SKAT-O. Age, sex, and *APOE*ε4 status were adjusted with the SKAT-O. Additionally, the “Chinese AD Exome” is the large whole-exome sequencing database of the Han Chinese population (Xu et al., 2018; Zhang et al., 2019a). The rare variants in our study were compared with those in the “Chinese AD Exome” available at www.alzdata.org (Xu et al., 2018).

#### Pathway-Based Association Test

We combined the rare variants in each pathway and performed the association test between sLOAD patients and cognitively healthy individuals using the SKAT-O test. SKAT-O was also used to adjust for age, sex, and *APOE*ε4 status.

A *P*-value of 0.05 was defined as the threshold for nominal significance. Moreover, Bonferroni corrections were performed for the common variant association test, gene-based association test, and pathway-based association test. A cutoff *P* value ^∗^ *n* < 0.05 was considered as statistically significant (n is defined by the number of common variants, genes, or pathways in the tests).

### Replication and Protein–Protein Interaction

The statistically significant common variants in our study were replicated in 30,344 LOAD patients and 52,427 cognitively healthy individuals of European ancestry from four consortia-conducted GWASs, which were meta-analyzed by the International Genomics of Alzheimer’s Project (IGAP). IGAP is a large two-stage study based upon GWASs on individuals of European ancestry. In stage 1, IGAP used genotyped and imputed data on 7,055,881 single-nucleotide polymorphisms (SNPs) to meta-analyze four previously published GWAS datasets consisting of 17,008 AD cases and 37,154 controls (The European Alzheimer’s disease Initiative-EADI, The Alzheimer Disease Genetics Consortium-ADGC, The Cohorts for Heart and Aging Research in Genomic Epidemiology consortium-CHARGE, The Genetic and Environmental Risk in AD consortium-GERAD). In stage 2, 11,632 SNPs were genotyped and tested for association in an independent set of 8,572 AD cases and 11,312 controls. Finally, a meta-analysis was performed combining results from stages 1 and 2 (Lambert et al., 2013). Additionally, protein–protein interactions (PPIs) were assessed using the STRING v11 database to determine whether the proteins involved in Aβ and tau metabolism were interacted with other AD-associated proteins using the STRING v11 database (Szklarczyk et al., 2019).

## Results

This study included 372 sLOAD patients and 345 cognitively healthy individuals, all of whom were of southern Han Chinese ancestry (Table 1).

### Common Variant Association Test

**FIGURE 2:**
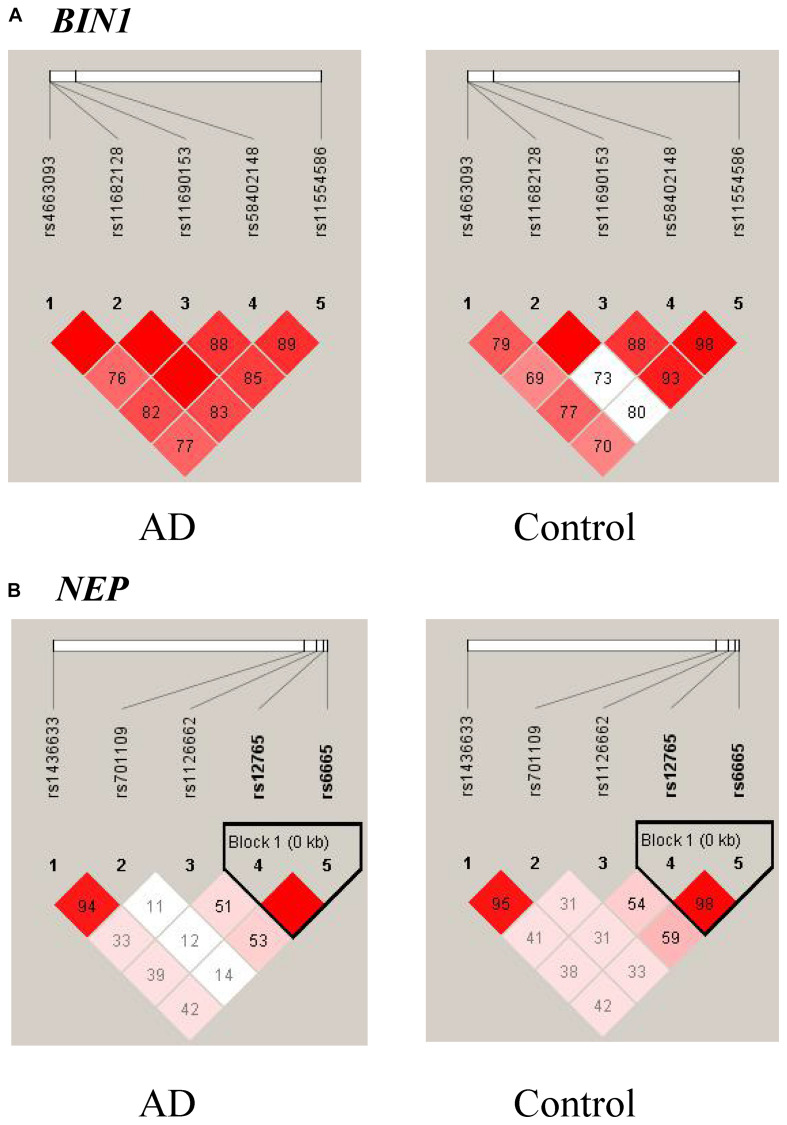
Linkage disequilibrium (LD) patterns of *BIN1* **(A)** and *NEP* **(B)**. The value in each square is equal to *r*^2^×100.

**FIGURE 3:**
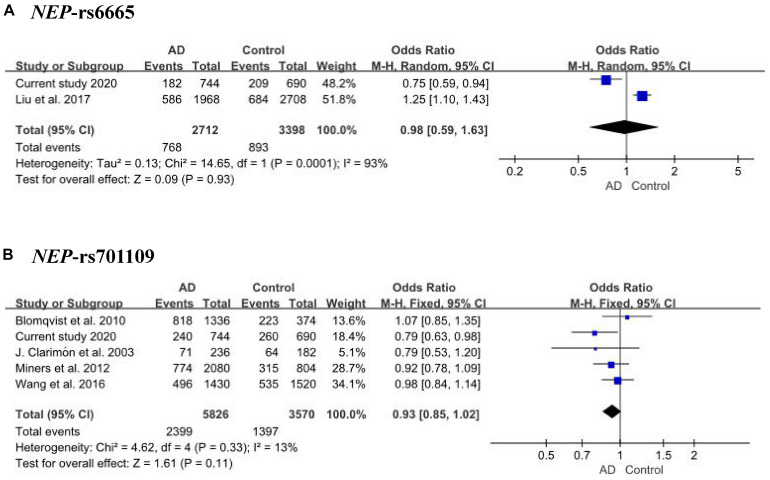
Meta-analysis of *NEP* rs6665 **(A)** and rs701109 **(B)** in our study and reported studies.

Ninety-eight variants remained after filtering out those with MAF ≤ 0.05, genotyping rate <80%, and Hardy–Weinberg *P* value < 0.001. We identified 13 nominally significant variants of genes involved in the metabolic pathways of tau pathology, tau phosphorylation, and Aβ degradation between sLOAD patients and cognitively healthy individuals (uncorrected *P* value < 0.05) (Table 2). Among the 13 nominally significant variants, six (46.2%) were UTR5 variants, four (30.8%) were UTR3 variants, two (15.4%) were intronic variants, and one (7.6%) was an exonic variant. SNP rs11682128 in *BIN1* reached statistical significance between sLOAD patients and cognitively healthy individuals after adjusting for age, sex, and *APOE* ε4 status and after performing the Bonferroni correction [corrected *P*-value = 0.000153, OR (95% CI) = 1.403 (1.079–1.824)], based on a corrected *P*-value [*P*-value < 0.00051 (0.05/98)] (Table 2). None of the common variants in the metabolic pathways of Aβ degradation, Aβ generation, tau kinases, and tau phosphatase differed significantly between sLOAD patients and cognitively healthy individuals after correction. The detailed information of the 98 variants is shown in Supplementary Table 1. The LD patterns of SNPs in the *BIN1* (rs11682128–rs11690153–rs11554586–rs58402148–rs4663093) and the *NEP* (rs1436633–rs1126662–rs701109–rs6665–rs12765) were similar between sLOAD patients and cognitively healthy individuals (Figure 2). Additionally, we performed the meta-analysis of nominally significant variants by using the data from our study and previous studies. We found that only *NEP* rs6665 and rs701109 were previously reported. No significant association was observed for *NEP* rs6665 and rs701109 in the meta-analysis of the current study and existing studies (Figure 3).

**TABLE 2:** Nominally significant common variants between sLOAD and cognitively healthy individuals.

Gene	Position	rs ID	Mutation regions	MAF (case/control)	*P*	Corrected *P*-value	OR	OR 95% CI
*BIN1*	chr2:127839474	rs11682128	UTR5	0.225/0.171	0.011	0.000153*	1.403	1.079–1.824
*BIN1*	chr2:127839534	rs11690153	UTR5	0.151/0.087	0.000	0.001795	1.861	1.335–2.595
*BIN1*	chr2:127864546	rs11554586	UTR5	0.134/0.087	0.004	0.001997	1.630	1.162–2.287
*BIN1*	chr2:127841945	rs58402148	UTR5	0.151/0.096	0.002	0.004478	1.675	1.212–2.316
*BIN1*	chr2:127839434	rs4663093	UTR5	0.163/0.217	0.008	0.022542	0.699	0.536–0.912
*PPP2CA*	chr5:133561589	rs3863186	UTR5	0.484/0.425	0.024	0.053867	1.270	1.031–1.565
*PPP2R2A*	chr8:26227640	rs3808565	Intronic	0.444/0.380	0.014	0.05845	1.302	1.054–1.608
*NEP*	chr3:154866453	rs1436633	Intronic	0.173/0.235	0.004	0.069732	0.684	0.528–0.886
*NEP*	chr3:154899943	rs1126662	UTR3	0.223/0.291	0.003	0.084561	0.699	0.551–0.887
*NEP*	chr3:154898407	rs701109	UTR3	0.323/0.377	0.031	0.178405	0.788	0.634–0.979
*MMP9*	chr20:44640225	rs17576	Exonic	0.242/0.293	0.030	0.261342	0.771	0.610–0.975
*NEP*	chr3:154901205	rs6665	UTR3	0.245/0.303	0.013	0.414257	0.745	0.590–0.941
*NEP*	chr3:154900690	rs12765	UTR3	0.237/0.293	0.016	0.530354	0.749	0.592–0.947

MAF, minor allele frequency; OR, odds ratio; CI, confidence interval. Corrected *P*-value, *P*-value after the adjustment of age, gender, and APOE ε4 status; UTR5, untranslated region 5; UTR3, untranslated region 3; sLOAD, sporadic late-onset Alzheimer’s disease. *Corrected *P*-value < 0.00051 (0.05/98) was considered as statistically significant.

### Gene-Based Association Test

In this study, the rare variants including coding and non-coding variants with MAF ≤ 0.05 were collapsed together, and their effects were studied between sLOAD patients and cognitively healthy individuals. After the Bonferroni correction was performed and age, sex, and *APOE* ε4 status were adjusted for, the rare variants of *NEP* and *FERMT2* reached nominal significance between sLOAD patients and cognitively healthy individuals (0.0026 < corrected *P*-value < 0.05) (Table 3). The detailed information of rare synonymous and non-synonymous variants is shown in Supplementary Table 2. Nine rare variants existed both in our study and in the “Chinese AD Exome” with similar frequencies. The results are added in Supplementary Table 3.

**TABLE 3:** Gene-based SKAT-O test.

Transcript ID	Position	Gene	Number of variants	P value	Corrected P value
NM_001397	chr1:21543823.21671981	*ECE1*	70	0.3996	0.1528
NM_033467	chr1:2522081.2564481	*MMEL1*	56	0.5486	0.3231
NM_001286126	chr1:220701525.220837799	*MARK1*	46	0.2011	0.2113
NM_139343	chr2:127805599.127864903	*BIN1*	54	0.0865	0.2066
NM_002093	chr3:119540800.119813264	*GSK3B*	70	0.8488	0.8357
NM_000902	chr3:154797436.154901518	*NEP*	60	0.0035	0.0232
NM_002715	chr5:133532148.133561950	*PPP2CA*	7	0.2398	0.2946
NM_004935	chr7:150750899.150755052	*CDK5*	15	0.8119	1
NM_002717	chr8:26149007.26230196	*PPP2R2A*	27	0.3782	0.2614
NM_001322795	chr10:94211441.94333852	*IDE*	55	0.2392	0.0729
NM_016147	chr11:73882108.73965748	*PPME1*	22	0.03766	0.2638
NM_012104	chr11:117156402.117186972	*BACE1*	18	0.4485	0.3147
NM_001134999	chr14:53323989.53417815	*FERMT2*	28	0.0192	0.0159
NM_006305	chr15:69070874.69113261	*ANP32A*	23	1	0.7828
NM_001032391	chr16:25123047.25189551	*LCMT1*	19	0.8202	0.9217
NM_000789	chr17:61554422.61575741	*ACE*	85	0.2817	0.2429
NM_006221	chr19:9945883.9960365	*PIN1*	15	0.2137	0.3725
NM_004994	chr20:44637547.44645200	*MMP9*	25	0.2139	0.3106
NM_138991	chr21:42539728.42654461	*BACE2*	84	0.2815	0.1322

*P value, *P* value before correction; Corrected *P*-value, *P*-value after the adjustment of age, gender, and *APOE*ε4 status; SKAT-O, optimized sequence kernel association test.*

*Corrected *P*-value < 0.0026 (0.05/19) was considered as statistically significant.*

### Pathway-Based Association Test

In addition to the gene-based association test, we performed the pathway-based association test between sLOAD patients and cognitively healthy individuals by combining the rare variants with MAF ≤ 0.05 within each pathway using the SKAT-O test. After adjustment for age, sex, and *APOE*ε4 status, the rare variants in the Aβ degradation, tau pathology, and tau phosphatase pathways were nominally significantly different between sLOAD patients and cognitively healthy individuals (0.01 < corrected *P*-value < 0.05). The rare variants in the Aβ generation and tau kinase pathways did not differ significantly between sLOAD patients and cognitively healthy individuals (corrected *P*-value > 0.05) (Table 4).

**TABLE 4:** Pathway-based SKAT-O test.

Pathway	Genes	Number of variants	*P*	Corrected *P* value
Aβ degradation	***NEP***, *IDE*, *MMP9*, *ACE*, *ECE1*, *MMEL1*	351	0.054	0.021*
	*IDE*, *MMP9*, *ACE*, *ECE1*, *MMEL1*	291	0.170	0.071
Aβ generation	*BACE1*, *BACE2*	102	0.252	0.098
Tau kinase	*GSK3B*, *CDK5*, *MARK1*	131	0.641	0.710
Tau pathology	***FERMT2***, *BIN1*	82	0.009	0.031*
	*BIN1*	54	0.087	0.207
Tau phosphatase	***PPME1***, *PPP2CA*, *PPP2R2A*, *PIN1*, *ANP32A*, *LCMT1*	113	0.017	0.033*
	*PPP2CA*, *PPP2R2A*, *PIN1*, *ANP32A*, *LCMT1*	91	0.206	0.129

In the Aβ degradation, tau pathology, and tau phosphatase pathways, the driver genes are in bold. *P* value, *P* value before correction; Corrected *P*-value, *P*-value after the adjustment of age, gender, and *APOE* ε4 status; SKAT-O, optimized sequence kernel association test.

*Corrected *P*-value < 0.01 (0.05/5) was considered as statistically significant.

### Replication and Protein–Protein Interaction

**FIGURE 4:**
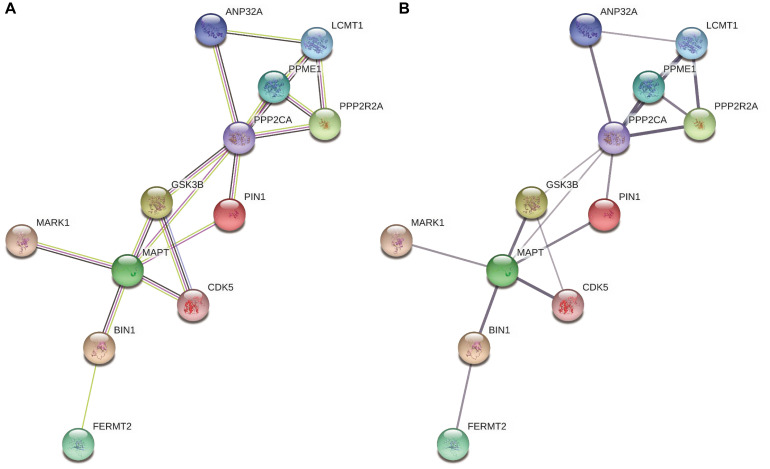
The interaction between BIN1 and other proteins involving the metabolic pathways of tau. **(A)** Colors represent different evidence sources (the experimental results, pink line; text mining sources, yellow line; gene co-expression, black line). **(B)** The thickness indicates the strength of protein interaction.

We replicated the *BIN1* rs11682128 based on 30,344 LOAD patients and 52,427 cognitively healthy individuals from the IGAP, which showed a significant association between *BIN1* rs11682128 and LOAD patients (*P* value: 1.451 × 10^–10^). PPI was performed to determine whether BIN1 was associated with proteins encoded by genes involved in the metabolic pathways of tau proteins (MAPT, GSK3B, CDK5, MARK1, FERMT2, PPP2CA, PPP2R2A, ANP32A, LCMT1, PPME1, and PIN1), which showed high confidence for the association between BIN1 and MAPT (Table 5 and Figure 4).

**TABLE 5:** The STRING interaction scores between BIN1 and other proteins.

Protein 1	Protein 2	Co-expression	Experimentally determined interaction	Text mining	Total scores
BIN1	MAPT	0.061	0.472	0.754	0.867
BIN1	FERMT2	0	0	0.641	0.641

*The total scores were equal to the different scores from different evidence sources (co-expression, experimental, and text mining), which were described in the STRING’s database. In this scoring system, 0.70 is the minimum required interaction score of high confidence between proteins.*

## Discussion

In this study, we systematically investigated whether the genes associated with Aβ and tau metabolism conferred genetic susceptibility to sLOAD patients in the southern Han Chinese population using targeted panel sequencing. The common variant association test revealed that SNP rs11682128 of *BIN1* increased the risk of sLOAD after adjusting for age, sex, and *APOE* ε4 status and performing the Bonferroni correction, which could be replicated in a European population from the IGAP. Furthermore, the rare variants of *NEP* and *FERMT2* as well as the Aβ degradation, tau pathology, and tau phosphatase pathways were nominally associated with the risk of developing sLOAD.

The abnormal accumulations of Aβ and tau proteins are pathological hallmarks of AD and contribute to the neurodegenerative process in the brains of patients with AD. Although tau deposition was considered a downstream event in the Aβ hypothesis, increasing evidence indicated that Aβ and tau protein accumulations lead to neurotoxicity in parallel (Gomes et al., 2019). Previous studies have identified that three pathogenic genes (*APP*, *PSEN1*, and *PSEN2*) are involved in the pathogenesis of EOAD, suggesting the significant role of Aβ metabolism in EOAD (Jiao et al., 2014). A recent GWAS found that several novel genome-wide risk loci are implicated in Aβ and tau metabolism in LOAD, suggesting that the genes involved in the metabolism of Aβ and tau were associated with both EOAD and LOAD (Kunkle et al., 2019). Given the potentially important roles of genes in the metabolism of Aβ and tau, several studies investigated the relationship of some of these genes with AD. SNP rs1816558 of *NEP* was associated with an increased risk of AD in the Han Chinese population (Wang et al., 2016a). In the Northern Han Chinese population, rs6665 of *NEP* elevated the risk of LOAD patients (Liu et al., 2017). SNP rs3781239 of *IDE* conferred susceptibility to AD in Han Chinese (Wang et al., 2012). *BIN1* is the second most important genetic risk factor in AD. SNP rs744373 of *BIN1* increased the risk of developing AD in populations from East China (Wang et al., 2016b). In young Chinese healthy individuals, *BIN1* rs744373 was linked with reduced functional connectivity and worse high-load working memory performance (Zhang et al., 2015). Nevertheless, in the Chinese population, studies on genes involved in the metabolic pathways of Aβ and tau remain limited. Most of the genetic data related to the metabolism of Aβ and tau are from the Caucasian population (Clarimon et al., 2003; Helisalmi et al., 2004; Wood et al., 2007; Miners et al., 2012; Sassi et al., 2016). Moreover, most of these studies focused on some SNPs by using array-based SNP genotyping. Thus, it is necessary to systematically investigate the association of genes involved in Aβ and tau metabolism with sLOAD in the Chinese population.

*BIN1* is located on chromosome 2q14.3 and encodes BIN1, a membrane deforming protein expressed primarily in the brain and muscle. BIN1 has a significant role in endocytosis, cytoskeleton regulation, DNA repair, and apoptosis (Prokic et al., 2014). BIN1 is also associated with tau pathology because it regulates endocytosis of the tau protein (Calafate et al., 2016). The BIN1 ortholog, Amph, mediates the risk of AD due to its involvement in tau neurotoxicity (Chapuis et al., 2013). A three-stage analysis of 8,371 AD cases identified that SNP rs744373 in *BIN1* attained genome-wide statistical significance (OR = 1.13; 95% CI:1.06–1.21; *P* = 1.6 × 10^–11^), suggesting that the *BIN1* gene is a risk factor for AD (Seshadri et al., 2010). In our previous study, we also found that the *BIN1* SNP rs744373 was associated with the risk of developing AD (Jiao et al., 2015). In addition, some GWASs showed that SNP rs6733839 in *BIN1* conferred susceptibility to AD (Lambert et al., 2013; Kunkle et al., 2019). A study revealed that *BIN1* SNP rs744373 was associated with memory deficit and higher tau deposits rather than amyloid deposition across brain regions using positron emission tomography–computed tomography scans, thus demonstrating that *BIN1* may lead to cognitive impairment through tau pathology *in vivo* (Franzmeier et al., 2019). In the present study, we found that SNP rs11682128 in *BIN1* was significantly associated with a higher risk of AD development and was replicated in the IGAP, which suggested that it can contribute to the risk of AD across different populations and may have a significant role in the development of AD. *BIN1* rs11682128 is located in the non-coding region. With the use of GenoCanyon, SNP rs11682128 in *BIN1* was predicted to be functional with a score of 1 (the cutoff value of the functional variant is 0.5) (Lu et al., 2015). Prioritization And Functional Assessment, a non-coding variant assessment software, showed that rs11682128 may be a functional variant (Zhou and Zhao, 2018). Additionally, by using another coding and non-coding variant assessment algorithm, Eigen-PC, the score of *BIN1* rs11682128 was 3.156, confirming that it is likely to be functional (Ionita-Laza et al., 2016). Subsequently, *BIN1* rs11682128 may be involved in the pathogenesis of AD via exhibiting regulatory activity in the expression of BIN1. Furthermore, there was high confidence regarding the interaction between BIN1 and MAPT, showing that BIN1 might exert a detrimental effect on AD by interacting with tau protein. Moreover, in our study, five common variants of the *NEP* gene were nominally significantly associated with sLOAD. Based on our data and those of the previously reported studies, the meta-analysis showed that *NEP* rs701109 and rs6665 did not differ significantly between AD patients and normal controls (Clarimon et al., 2003; Blomqvist et al., 2010; Miners et al., 2012; Wang et al., 2016a; Liu et al., 2017). Larger studies are warranted to elucidate the role of these two SNPs in the pathogenesis of AD.

High-throughput sequencing has made it possible to ascertain nearly all genetic variations, including rare variants, which are defined as variants with a MAF < 1–5%. Despite its potential importance, a rare variant would have a limited impact on a certain disease owing to its low allele frequency (Neale et al., 2011). Thus, there is a need to develop new methods to analyze rare variants by combining information on rare variants in genes or pathways. The burden test was the commonly used method for evaluating rare variants, in which all rare variants were summarized as a single genetic variable and subsequently tested for association with the disease. However, the burden test is limited in cases where either several neutral variants or both protective and risk variants are present (Morris and Zeggini, 2010). To overcome these limitations, another method, the SKAT, was proposed to evaluate the associations for rare variants (Wu et al., 2011). Nevertheless, the power of the SKAT is lower than that of the burden test if the rare variants impact a certain disease in the same direction. Lee et al. (2012) established the SKAT-O test, a linear combination of the burden test and SKAT, thus maximizing the power for analyzing rare variants. SKAT-O is widely used in neurological diseases, including AD (Nho et al., 2017), Parkinson’s disease (Shu et al., 2019), and cerebrovascular disease (Liao et al., 2019).

The imbalance between production and degradation of Aβ leads to the formation of senile plaques, which have deleterious effects on brain regions, especially the temporal cortex (Blomqvist et al., 2010). Previous studies have identified that variants of genes involved in the degradation of Aβ were associated with AD. SNPs rs989692 and rs3736187 in *NEP* differed significantly between AD and cognitively healthy individuals in a Finnish population, suggesting that *NEP* could increase the risk of AD (Helisalmi et al., 2004). The rare variants of *ECE1* were nominally associated with AD after Bonferroni correction, indicating that *ECE1* may increase the susceptibility to AD (Sassi et al., 2016). We previously identified that another novel Aβ degradation gene, *ECE2*, was a risk factor for AD development (Liao et al., 2020). In this study, the rare variants of *NEP* and Aβ degradation pathway were nominally significantly different between sLOAD and cognitively healthy individuals, which indicates the potentially significant roles of the rare variants of Aβ degradation pathway in the pathogenesis of sLOAD.

The fly orthologs of FERMT2 (fit1/fit2) were identified as tau modifiers in a *Drosophila* model of AD (Shulman et al., 2014). SNP rs17125944 in *FERMT2* was associated with tau pathology in patients with AD (Farfel et al., 2016). The gene-based association test revealed that the non-synonymous variants of *FERMT2* were also associated with LOAD (Beecham et al., 2018). The loci of the *BIN1* gene were significantly associated with cerebrospinal fluid T-tau and P-tau levels, suggesting that *BIN1* plays an important role in tau pathology (Wang et al., 2016c). Moreover, different GWASs identified that *BIN1* and *FERMT2* increased the susceptibility to LOAD (Sun et al., 2017). The rare variants of *FERMT2* and the pathway of tau pathology reached the nominal significance in our study, further suggesting the significance of genes involved in tau pathology in the pathogenesis of sLOAD.

A decrease in PP2A levels is associated with tau hyperphosphorylation. Immunoblotting analysis of AD autopsy cases revealed a significant decrease of PP2A levels in the frontal and temporal cortices (Sontag et al., 2004). Vázquez-Higuera et al. (2011) found that several SNPs of genes involved in the PP2A pathway, including *PPP2CA*, *PPP2R2A*, *ANP32A*, *LCMT1*, *PPME1*, and *PIN1*, were not related to the risk of AD. However, in the Chinese population, SNP rs2287839 in the *PIN1* promoter was significantly associated with the delayed onset of AD (Ma et al., 2012). Our study revealed that the rare variants in the pathway of tau phosphatase were nominally significantly correlated with sLOAD, suggesting their potentially important role in the development of sLOAD. In addition, we found that the frequencies of rare variants associated with Aβ and tau metabolism were similar between our study and the “Chinese AD Exome” (Xu et al., 2018; Zhang et al., 2019a). This indicates that rare variants exhibited high consistency in the Chinese population.

In summary, our study revealed that SNP rs11682128 in *BIN1* was significantly associated with the risk of sLOAD, suggesting its significance in sLOAD development. Besides, the rare variants of *NEP* and *FERMT2* as well as the pathways of Aβ degradation, tau pathology, and tau phosphatase were nominally associated with sLOAD, indicating that they may be the important risk factors for sLOAD.

## Data Availability Statement

The raw data supporting the conclusions of this article will be made available by the authors, without undue reservation.

## Ethics Statement

The studies involving human participants were reviewed and approved by the Ethics Committee of Xiangya Hospital of the Central South University. The patients/participants provided their written informed consent to participate in this study.

## Author Contributions

XX, BJ, and LS: study design, acquisition of data, analysis and interpretation of data, and drafting/revising the manuscript. XLia, WZ, ZY, and LG: analysis and interpretation of data. XW, LZ, and XLiu: collecting patients and clinical assessment. XY and BT: review and editing the manuscript. All authors contributed to the article and approved the submitted version.

## Conflict of Interest

The authors declare that the research was conducted in the absence of any commercial or financial relationships that could be construed as a potential conflict of interest.

## Footnotes

1 http://bio-bwa.sourceforge.net

2 http://broadinstitute.github.io/picard/

3 https://software.broadinstitute.org/gatk/

4 https://hpc.nih.gov/apps/ANNOVAR.html

5 http://www.hgvs.org/

6 https://training.cochrane.org/online-learning/core-software-cochrane-reviews/revman/revman-5-download
