# Supplementary material for: Association of Genes Involved in the Metabolic Pathways of Amyloid-β and Tau Proteins With Sporadic Late-Onset Alzheimer’s Disease in the Southern Han Chinese Population
Source: Front Aging Neurosci. 2020 Nov 6;12:584801. doi: 10.3389/fnagi.2020.584801 (PMC7677357; doi:10.3389/fnagi.2020.584801)
Supplement: Supplementary file 3 [file Table_3.docx]

Supplementary table 3. Rare variants existed both in our study and in the “Chinese AD Exome”

| **Gene** | **Variant** | **Current study** | | | | **Chinese AD exome** | | | |
| --- | --- | --- | --- | --- | --- | --- | --- | --- | --- |
|  |  | **AD** | **Control** | **P** | **OR** | **AD** | **Control** | **Fisher_P** | **Fisher_OR** |
| *ACE* | rs117134739 | 19/744 | 14/690 | 0.507847* | 1.265 | 6/212 | 14/736 | 0.417564 | 1.50208 |
| *ECE1* | rs367812436 | 2/744 | 0/690 | 0.500361 | 1.929919 | 1/208 | 0/366 | 0.362369 | 5.2988 |
| *ECE1* | rs576971913 | 4/744 | 1/690 | 0.375903 | 3.724324 | 1/212 | 1/366 | 1 | 1.72986 |
| *IDE* | rs117251230 | 20/744 | 7/690 | 0.030565* | 2.695343 | 3/212 | 10/736 | 1 | 1.04211 |
| NEP | rs200308077 | 1/744 | 0/690 | 1 | 1.928668 | 2/214 | 0/366 | 0.135733 | 8.62353 |
| BIN1 | rs141119288 | 22/744 | 10/690 | 0.053443* | 2.072022 | 7/204 | 7/734 | 0.0176092 | 3.69036 |
| BIN1 | rs117721706 | 19/744 | 9/690 | 0.087539* | 1.982989 | 7/214 | 16/736 | 0.446523 | 1.52174 |
| LCMT1 | chr16:25186310 | 1/744 | 0/690 | 1 | 1.928668 | 1/212 | 2/320 | 1 | 0.753555 |
| PPME1 | rs190849735 | 6/744 | 3/690 | 0.509240 | 1.862 | 1/212 | 6/736 | 1 | 0.576619 |

*:P value was calculated by chi-square test;
